# Early pathological changes in the liver and kidney of non-obese diabetic (NOD) mice: involvement of iron accumulation and ferroptosis

**DOI:** 10.3389/fendo.2025.1673012

**Published:** 2025-10-02

**Authors:** Ana Stancic, Milica Markelic, Nevena Savic, Ksenija Velickovic, Vesna Martinovic, Andjelija Gudelj, Danica Velickovic, Ilijana Grigorov, Vesna Otasevic

**Affiliations:** ^1^ Department of Molecular Biology, Institute for Biological Research “Siniša Stanković”, National Institute of Republic of Serbia, University of Belgrade, Belgrade, Serbia; ^2^ Department of Cell and Tissue Biology, University of Belgrade – Faculty of Biology, Belgrade, Serbia; ^3^ Blood Transfusion Institute of Serbia, Belgrade, Serbia

**Keywords:** ferroptosis, iron accumulation, diabetic nephropathy, liver pathology, NOD mice

## Abstract

Disorders of iron metabolism and ferroptosis play an important role in the development of diabetes and related pathologies. The involvement of ferroptosis in type 1 diabetes has mainly been investigated in animal models with chemically induced diabetes. Our aim was to examine the involvement of iron homeostasis disturbances and ferroptotic events in liver and kidney damage in non-obese diabetic (NOD) mice in the early phase of spontaneous development of diabetes (15 days of stable hyperglycemia). We found an accumulation of iron and lipid peroxides in the proximal tubule epithelial cells (PTECs) of the renal cortex and in the liver. This was accompanied by a decrease in the level of proteins involved in the sequestration (ferritin) and export (ferroportin) of iron and an increase in the level of transferrin receptor 1 in both organs. The level of activated nuclear factor erythroid 2-related factor 2 was decreased in both liver and kidney, whereas lower levels of Xc- glutamate/L-cystine antiporter and glutathione peroxidase 4 were detected only in PTECs, demonstrating the proferroptotic events in these cells. In conclusion, although iron accumulation and lipid peroxidation occur in both organs, the kidneys are more susceptible to ferroptosis in early diabetes development.

## 1 Introduction

Diabetes is a complex metabolic disease with an increasing epidemiological trend worldwide. Chronic or intermittent hyperglycemia in diabetes is considered to be the main cause of the development of diabetic complications. It leads to overproduction of reactive oxygen species (ROS) and induces oxidative stress, leading to cell damage and cell death in various organs, as summarized previously (1). Hyperglycemia can also lead to an overload of labile iron ions and dysregulation of iron homeostasis (2). Recently, it has been recognized that one of the most important mechanisms of diabetes-related cell death is ferroptosis (3–6). This type of regulated cell death is related to the accumulation of lipid peroxides, products of polyunsaturated fatty acids (PUFAs) damage, which is mainly due to the overload of labile iron ions and ROS (7, 8). Lipid peroxidation is controlled by antioxidative enzymes, with glutathione peroxidase 4 (GPX4) playing the most important role. It catalyzes the removal of lipid peroxides using glutathione (GSH) as a cofactor. The GSH pool in the cell depends on the activity of the Xc- glutamate/L-cystine antiporter (SLC7a11, xCT), which is another important player that determines whether the cell undergoes ferroptosis or remains viable.

There is increasing evidence from our team and others that impaired iron metabolism and ferroptosis are involved in the etiology and pathogenesis of diabetes and diabetic complications, including liver pathology and diabetic nephropathy (DN) (4, 5, 9). Most of these data on the role of ferroptosis in diabetic complications come from animal studies of chemically induced type 1 diabetes (T1D) or high-fat diet-induced type 2 diabetes (T2D). As far as we are aware, there is no information on this topic in non-obese diabetic (NOD) mice, although this is currently the most suitable animal model for T1D (10). In contrast to chemically induced diabetes, in which the diabetogenic agents (streptozotocin or alloxan) predominantly destroy β-cells directly, disease in this polygenic models of diabetes develops through the spontaneous infiltration of the immune cells into the pancreatic islets and the consequent destruction of the β-cells (11). Diabetes in NOD mice has many pathological and genetic similarities to T1D in humans (12), and as with T1D in humans, chronic hyperglycemia leads to damage to the kidney, liver, nerves, retina, heart, etc. if left untreated. All this makes the NOD mice the gold standard for preclinical investigation of the etiology, pathology and progression of T1D and its complications (13, 14). These studies have contributed enormously to our understanding of human T1D. Yip et al. (15) recently suggested that the disruption of iron homeostasis in the pancreatic islets of NOD mice may occur during the onset of “destructive” insulitis. Interestingly, they showed that the severity of NOD disease correlates positively with dietary iron intake. Conversely, diabetes can promote systemic iron loading (16), but the mechanism is not yet well understood.

Diabetic complications usually occur in experimental animals after prolonged hyperglycemia, with onset and progression varying depending on the specific complication and animal model used. Pathological changes in the liver of diabetic mice usually begin 2–4 weeks after the onset of hyperglycemia and include changes such as reduced glycogen synthesis due to insulin deficiency and excessive gluconeogenesis in hepatocytes, which promote hepatic lipolysis and ketogenesis (17), oxidative stress, mild steatosis and inflammation (18, 19). A few data on liver pathology are available in NOD mice, showing altered liver enzyme activity, inflammation and damage (20). DN usually develops 4–6 weeks after the onset of hyperglycemia, while severe damage occurs after 3–6 months (21, 22). In NOD mice, the data are similar, showing the development of albuminuria, a marked increase in glomerular filtration rate, and glomerular morphological changes at an early stage of diabetes (21 days of hyperglycemia) (23–25). However, in this model, there is no detailed information on the pathological changes in the epithelial cells of the cortical tubules (tubulocytes) and the underlying mechanisms, especially related to lipid peroxidation and ferroptosis. Since diabetes inducers such as streptozotocin (STZ) are also known to have direct cytotoxic effects on hepatocytes and renal tubulocytes (26, 27), analyzing the changes in animals that spontaneously develop diabetes could help to determine the actual effects of hyperglycemia.

In the present study we focused on the pathological changes in the liver and kidney of NOD mice associated with early diabetes. The main focus was on possible disturbances of iron metabolism and the involvement of ferroptosis in hepatocyte and renal tubulocytes’ damage. These data could pave the way for timely and appropriate intervention to prevent the development of severe nephropathy and liver disease in diabetic patients.

## 2 Materials and methods

### 2.1 Experimental design

This is an observational study of disease progression in NOD mice. The comparison animals consisted of sex-matched (female) disease-free NOD/ShiLtJ mice (strain #001976, The Jackson Laboratory, USA; local supplier Charles River, Italy) that did not receive any experimental treatments and were kept and monitored in the same facility of the Institute for Biological Research “Siniša Stanković”, University of Belgrade. All animals were handled and examined according to the same scheme (including blood glucose measurement, weighing, and other procedures). The mice were housed under identical conditions (specific pathogen-free facility - Uniprotect Air Flow Cabinet (Zoonlab, Germany), 12-hour light/dark cycle, water *ad libitum*). Baseline data (age, body weight, baseline glucose) were recorded and used to assess the comparability of the groups. All experiments were approved by the Ethics Committee of the Institute for Biological Research “Siniša Stanković” (App. No. 323-07-05815/2020-05/1) following the Directive 2010/63/EU. Only female mice were used, as the development of diabetes is more predictable in female NOD mice (28). Blood glucose levels were measured weekly from the tenth week of life of the mice using an Accu-Check glucometer (Accu-Check Performa, Roche Diabetes Care, Germany) from the tail vein. The animals with a blood glucose level of over 12 mmol/l were classified as diabetic. These animals were examined after two weeks of hyperglycemia and were considered the early diabetic group. The control animals were normoglycemic, approximately 10-week-old NOD mice. The animals were euthanized by cervical dislocation between 9:00 and 9:30 am. Pancreas, liver and kidney samples were collected and routinely processed for microscopic, immunoblot and spectrophotometric analyses.

### 2.2 Sample size and statistical power

We used n = 8 mice per group. For the primary endpoint (glycemia), a sensitivity analysis was performed. With two-sided α = 0.05 and equal group sizes (n = 8), a two-sample t-test has 80% power to detect a standardized mean difference of Cohen’s d ≈ 1.51. Using the pooled SD from our data (σ = 3.30 mmol/L), this corresponds to a minimum detectable raw difference of Δ_MDES ≈ 4.97 mmol/L. Thus, the study was powered to detect large effects. For transparency, we report effect sizes with 95% confidence intervals (CIs) for the primary endpoint, whereas secondary/exploratory outcomes are summarized as mean ± SEM. For glycemia, the observed mean difference was 21.49 mmol/L, with a 95% CI [17.96, 25.03] mmol/L. Precision of estimation can be summarized by the CI half-width (H ≈ 3.54 mmol/L). Other readouts (histological, biochemical, and molecular parameters) were analyzed as secondary/exploratory outcomes.

### 2.3 Biochemical analysis

After blood isolation, serum was prepared and stored at -80 °C until further analysis. Serum urea and creatinine levels were measured spectrophotometrically (INEP, Serbia). For the biochemical analysis, part of the liver tissue was prepared in PBS (0.01 M, pH 7.4). Kinetic assays of alanine aminotransferase (ALT) and aspartate aminotransferase (AST) activity in liver tissue were determined spectrophotometrically (Shimadzu UV-160 spectrophotometer, Japan) using Bioanalytica kits (ALT-250 and AST-250, respectively, Bioanalytica, Serbia).

### 2.4 Microscopic examination

For the microscopic analyses, central portion of the pancreas, one kidney and the right median lobe of the liver were dissected immediately after the animals were euthanized. After cutting into halves (kidney) or slices (liver), the organs were placed in 10% neutral buffered formalin for 24 hours, dehydrated in graded ethanol solutions and cleared in xylene before embedding in paraffin (Histowax, Histolab, Sweden). After routine deparaffinization and rehydration, the 5 mm thick sections were used for histological, histochemical and immunofluorescence analyses.

#### 2.4.1 Heidenhain’s AZAN trichrome staining

To determine the presence/stage of fibrosis in the tissue of renal cortex and liver parenchyma, AZAN trichrome staining was performed as previously described (9). Fibrosis, reflecting extensive collagen deposition, is visible as intense blue staining and was scored in the liver tissue as follows: 0 – absent, 1 – mild, 2 – moderate, 3 – massive, and the mean values per group were indicated.

#### 2.4.2 Periodic acid Schiff staining

PAS staining is used for both kidney and liver samples and was performed as we have already described (9). In renal tissue, PAS is used to detect the basal lamina of the tubular epithelium and the glomerular capillary loops, while in hepatocytes PAS allows the detection of glycogen deposition. Glycogen deposits and glycosylated components of the basal lamina/glycocalyx are detectable as magenta-colored structures.

#### 2.4.3 Pearl’s staining - iron detection

To detect unbound iron (Fe^3+^) ions in liver and kidney tissue, Pearl’s histochemical staining was performed as described in our previous work (6). A positive reaction is visible as blue intracellular staining. To quantify iron loading in liver tissue and renal PTECs, the color deconvolution tool in Image J (National Institutes of Health, USA) was used (FastRed-FastBlue-DAB setup), and images with blue signal were used to determine the mean grayscale value of liver tissue and PTECs per group. Arbitrary units were calculated as a subtraction of the obtained grayscale values from 255 to obtain the values directly proportional to the signal intensity.

#### 2.4.4 Immunohistochemistry

Routine immunohistochemical analysis was performed on pancreatic, renal and liver tissue sections of three animals per group. Pancreatic tissue was analyzed for the insulin (rabbit anti-insulin, 1:100, sc-9168, Santa Cruz Biotechnology, USA), while liver and kidney were analyzed for the immunopositivity to: 4-hydroxy-2-nonenal (rabbit anti-4-HNE, 1:500, ab46545, Abcam, UK), cleaved caspase-3 (rabbit anti-cleaved Cas-3, 1:1000, 9664s, Cell Signaling Technology, USA), ferritin heavy chain 1 (mouse anti-FTH1, 1:100, sc-376594, Santa Cruz Biotechnology), transferrin receptor 1 (rabbit anti-TFR1, 1:200, ab840361, Abcam), ferroportin (rabbit anti-FPN, 1:200, PA5-22993, Thermo Fisher Scientific, USA), hepcidin (rabbit anti-hepcidin, 1:100, ab190775, Abcam), xCT (goat anti-xCT, 1:100, sc-79360, Santa Cruz Biotechnology), GPX4 (rabbit anti-GPX4, PA5-102521, Thermo Fisher Scientific, USA), nuclear factor erythroid 2-related factor 2 (rabbit anti-Nrf2, 1:60, ab31163, Abcam) and ferroptosis suppressor protein 1 (mouse anti-FSP1, 1:100, sc-377120, Santa Cruz Biotechnology). Secondary antibodies used were: goat anti-rabbit (1:1000, ab97051, Abcam), horse anti-mouse (7076S, 1:1000, Cell Signaling Technology) and donkey anti-goat (1:250, ab97770, Abcam).

After deparaffinization and rehydration, the antigen retrieval in citrate buffer, followed by blocking of the endogenous peroxidase (in 10% H_2_O_2_-methanol solution) was performed. After washing in phosphate-buffered saline (PBS), non-specific antibody binding was prevented by incubating the sections in a suitable serum protein solution (10% normal goat serum or 5% bovine serum albumin (BSA)). After addition of the primary antibody solution in 1% BSA, the samples were incubated overnight at 4 °C. After thorough rinsing in PBS, the samples were incubated with the corresponding secondary antibody for 1 hour at room temperature. The rinsed samples were then counterstained with Mayer’s hematoxylin, dehydrated and mounted in dibutyl phthalate polystyrene xylene (DPX) medium (Sigma Aldrich, USA).

The quantification of immunopositivity of liver tissue for: 4-HNE, cleaved Cas-3, FSP1, FPN and hepcidin, and of PTECs/DTECs for: 4-HNE, cleaved Cas-3, GPX4, xCT, FSP1 and hepcidin was performed similarly to Pearl staining - the color deconvolution tool in Image J was applied with an H-DAB setup and images with DAB signal were used to determine the mean grayscale value per group, which was subtracted from 255. The arbitrary units thus obtained were plotted. Nuclear immunopositivity of hepatocytes for Nrf2 was scored as follows: 0 – absent, 1 – low, 2 – high, and the mean values per group were indicated.

#### 2.4.5 TUNEL and propidium iodide staining

To analyze the presence of DNA fragmentation associated with cell death, fluorescent TUNEL staining (*In Situ* Cell Death Detection Kit, Fluorescein; Roche Applied Science, Germany) of liver and kidney tissue sections was performed as previously described (6). DNase-treated tissue sections served as positive control. Counterstaining with propidium iodide (PI) was performed to analyze the degree of nuclear condensation. Samples were analyzed using the SP5 confocal microscope (Leica Microsystems, Germany). The average TUNEL and PI fluorescence intensity of hepatocytes’ and PTECs/DTECs’ nuclei was measured using LAS AF software (Leica Microsystems).

### 2.5 Analysis of SOD and GPX activity in the liver and kidney tissue

To investigate the antioxidative defense system, the kidney and liver tissues were excised and thoroughly rinsed with saline to remove traces of blood. To measure superoxide dismutase (SOD) activity in the tissues, 10% homogenates of kidney and liver prepared in sucrose buffer (0.25 M sucrose, 0.1 mM EDTA and 50 mM Tris-HCl pH 7.4) were used. The total activity of SOD was determined according to the method described by Misra and Fridovich (29), but at 26 °C and expressed in units mg^−1^ of protein. SOD units were defined as the amount of the enzyme that inhibits the auto-oxidation of epinephrine under the appropriate reaction conditions. The activity of GPX was determined spectrophotometrically using t-butyl hydroperoxide as a substrate (30) and expressed in nmol of reduced NADPH min-1 mg-1 proteins.

### 2.6 SDS-Polyacrylamide Gel Electrophoresis (PAGE) and Western blot analysis

For SDS-PAGE and Western blot analysis, homogenates of kidney and liver tissue prepared in sucrose buffer with protease and phosphatase inhibitors (Protease Inhibitor Mix G, #39101, Serva Electrophoresis, Heidelberg, Germany) were used as previously described (3). For western blot analysis, eight homogenates from each group were pooled by two, thus obtaining four samples per group. Protein content in the samples was estimated by the method of Lowry et al. [62]. Ten μg of total protein extracts was separated by electrophoresis in 12% SDS-PAGE, transferred onto polyvinylidene fluoride (PVDF) membranes (10600023, Amersham Hybond P 0.45 PVDF, GE Healthcare Life Sciences, UK), and blocked in TBST solution (0.2% Tween 20, 50 mM Tris-HCl pH 7.6, 150 mM NaCl) containing 3% bovine serum albumin. Membranes were then incubated overnight with the following rabbit primary antibodies: anti-FTH1 (1:1000, #3998, Cell Signaling Technology), anti-phospho-acetyl-CoA carboxylase 1 (pACC1, 1:1000, #3661), anti-xCT (1:1000, CST#12691), all from Cell Signaling Technology, anti-GPX4 (1:1000, ab125066), anti-FPN (1:1000, ab78066), β-actin (1:2000, ab8227), purchased from Abcam and anti-TFR1 (1:1000, 13-6800) and phospho-Nrf2 (pNrf, 1:1000, PA5-67520), purchased from Thermo Fisher Scientific. After incubation with primary antibodies, membranes were probed with anti-rabbit HRP-conjugated secondary IgG antibodies (1:4000; ab6721, Abcam). Detection of immunoreactive bands was performed by an enhanced chemiluminescence detection system (sc-2048, Santa Cruz Biotechnology) using an iBright CL1500 Imaging System (Thermo Fisher Scientific). Quantitative analysis of immunoreactive bands was conducted densitometrically by ImageJ software (National Institutes of Health) (3). The ratio of dots per band for the target protein and β-actin (gel loading control) from three independent experiments was averaged, and changes in protein level were expressed as a percentage of an untreated control sample, which was standardized as 100%.

### 2.7 Statistical analysis

Statistical analyses were performed using GraphPad Prism software (GraphPad, USA). For most analyses, two means were compared using Student’s t-test. When more than two means were compared, the Kolmogorov-Smirnov test was used to test the data for normality. When the F-test indicated an overall difference, a one-way analysis of variance (one-way ANOVA) was performed (for the comparison of glycaemia level), except when the immunopositivity of PTECs and DTECs was compared within group and between groups, then a two-way ANOVA was performed. The Tukey multiple comparison test was used in both analyses. In all cases, statistical significance was set at p < 0.05.

## 3 Results

### 3.1 Early development of diabetes in NOD mice

**Figure 1:**
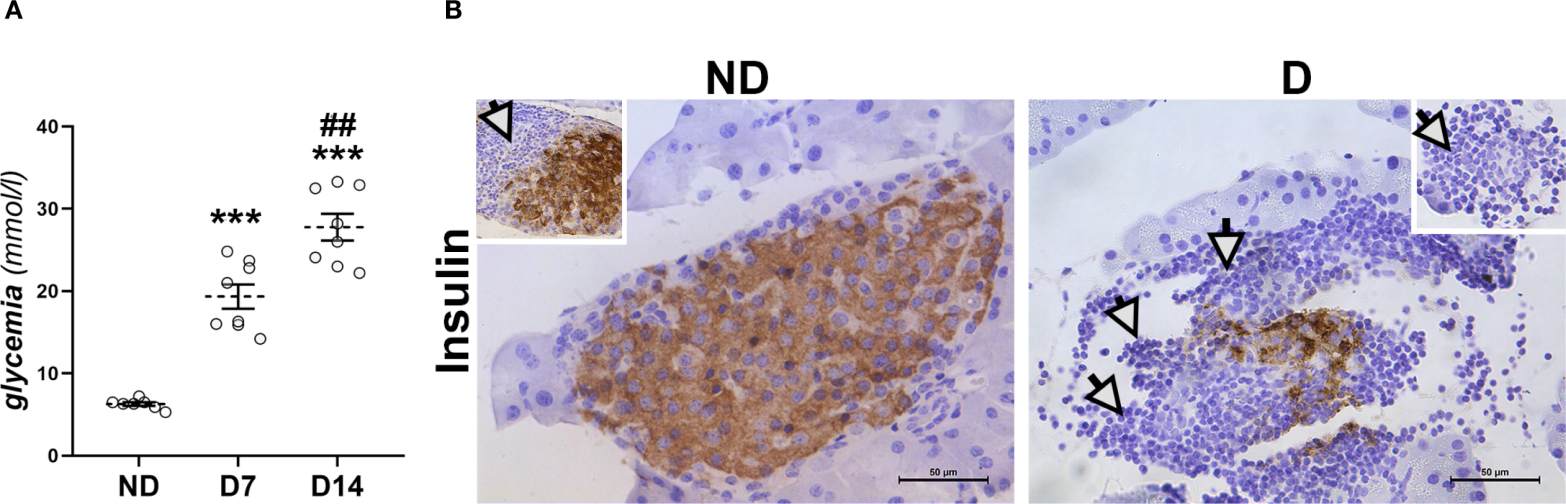
Development of diabetes in NOD mice. **(A)** Glucose serum levels in non-diabetic animals (ND) and in animals that were diabetic for 7 and 14 days (D7, D14). Values are expressed as mean ± SEM. Statistical significance compared to ND group (***p<0.001) and D7 (^##^p<0.01). **(B)** Immunohistochemical detection of insulin-positive β-cells in islets of Langerhans from ND mice and mice that were diabetic for 14 days (D). White arrows – immune cell infiltration around islets. Original magnification – x40, scale bar – 50 µm.

As shown in **Figure 1A**, the average blood glucose level in NOD mice gradually increases, reaching 18.9 mmol/l seven days (D7) after the first hyperglycemic value and rising to 27.7 mmol/l one week later (D14). To confirm the development of diabetes, immunohistochemical analysis of insulin-positive β-cells in the islets of Langerhans of the diabetic animals was performed (**Figure 1B**). These results showed a significant deterioration of the endocrine pancreas on D14, as only a small number of insulin-positive cells were detectable. The islets are rare and severely affected by (peri)insulitis, which can be recognized by the surrounding and infiltrating immune cells. It is worth noting that even in NOD mice without detectable hyperglycemia (ND group), in which islets with strong insulin positivity of numerous β-cells were preserved, periinsulitis is frequently observed.

The biochemical parameters were evaluated to assess the renal and hepatic function of the diabetic NOD mice (**Table 1**). Serum creatinine and urea levels were significantly increased in the diabetic animals (p<0.05). The activity of the liver enzyme alanine aminotransferase (ALT) was also increased in the diabetic animals (p<0.05), while the activity of aspartate aminotransferase (AST) remained statistically unchanged, despite the tendency to increase.

**Table 1:** Biochemical parameters of kidney and liver functional status.

Parameters	ND	D
Serum Urea (mmol/l)	3.66 ± 0.27	4.72 ± 0.31^*^
Serum Creatinine (µmol/l)	41.36 ± 4.4	66.34 ± 6.7^*^
Liver ALT (U/mg protein)	3750.8 ± 181.9	4780.9 ± 349.4^*^
Liver AST (U/mg protein)	22081.01 ± 116.5	25814.3 ± 1522.7

ND, non-diabetic group; D, diabetic group. Data are shown as mean ± SEM. Statistical significance: compared with the ND group (*), * p < 0.05.

### 3.2 Early histopathological changes in the liver of diabetic mice

**Figure 2:**
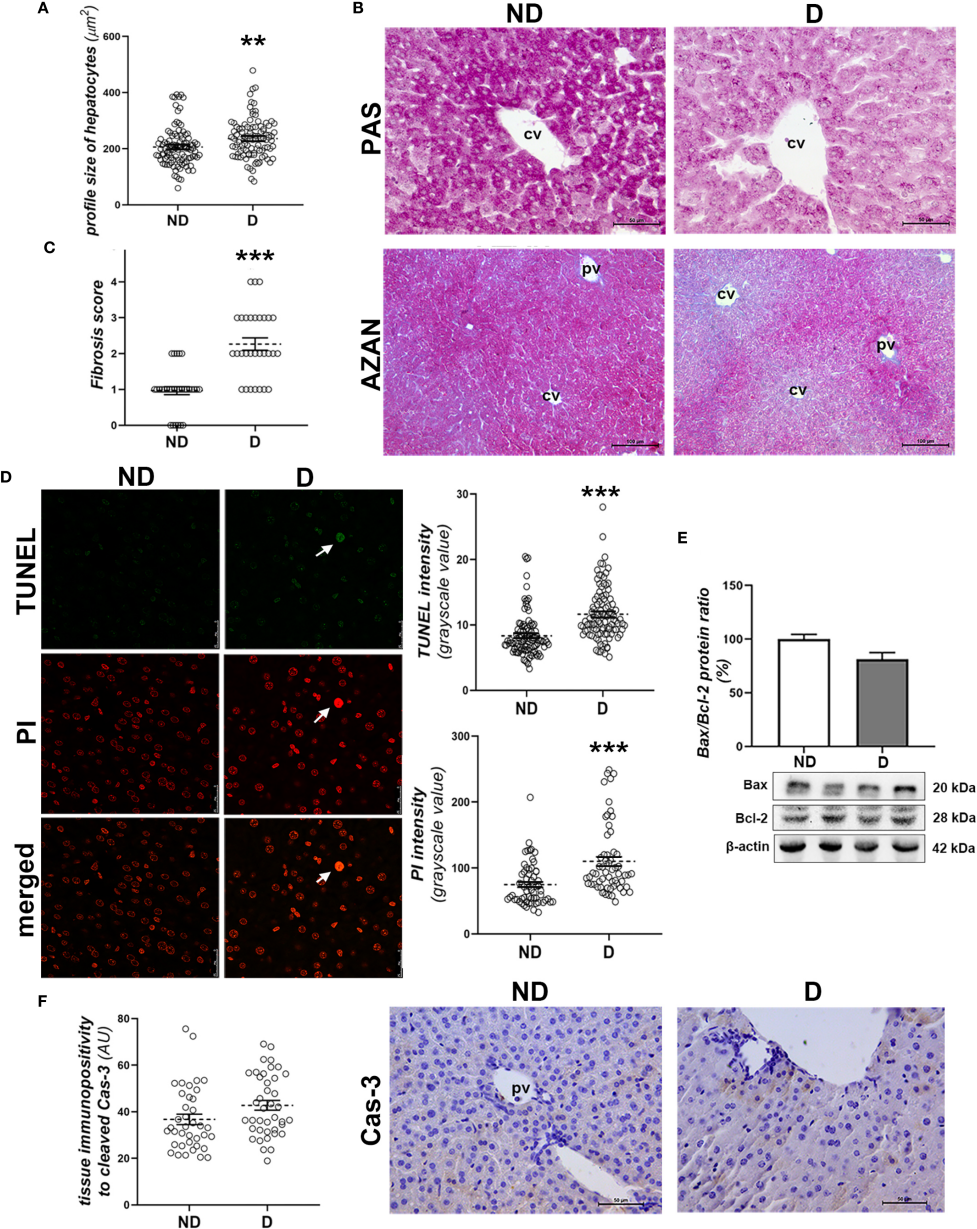
Early diabetic histopathological changes, cell death and damage in the liver of NOD mice. **(A)** Profile size of hepatocytes; **(B)** glycogen detection (magenta) - PAS staining; **(C)** fibrosis scoring and collagen detection (blue) in the liver of non-diabetic (ND) and **(D)** animals - AZAN staining; **(D)** TUNEL and PI staining and subsequent quantification of these fluorescent signals in the nuclei of hepatocytes; **(E)** comparison of hepatic Bax/Bcl-2 ratio (%); **(F)** quantification of the tissue immunopositivity and immunohistochemical detection of cleaved caspase-3 (Cas-3). All graph values are mean ± SEM. Statistical significance compared to the ND group: **p<0.01, ***p<0.001. Original magnification and scale bars: **(B, F)** x40, 50 μm, **(C)** x20, 100 μm; **(D)** x63, 25 μm. Markings on the micrographs: cv, centroportal vein; pv, portal vein; white arrows, late apoptotic nuclei; strongly TUNEL and PI positive.

Histopathological analysis of the liver showed signs of changes typical of diabetes in NOD mice that were hyperglycemic for 2 weeks. Swollen hepatocytes (**Figure 2A**) with decreased glycogen accumulation (**Figure 2B**) and mild fibrosis surrounding the central and portal veins (p<0.001; **Figure 2C**) are indicative of the early pathological changes.

TUNEL and PI staining showed increased DNA fragmentation (p<0.001; **Figure 2D**, TUNEL staining quantification) and chromatin condensation in diabetic animals (p<0.001; **Figure 2D**, PI staining quantification). Still, hepatocytes with pyknotic and strongly TUNEL-positive nuclei were rare (**Figure 2D**). Molecular analysis of the involvement of apoptosis in early diabetic deterioration revealed an unchanged Bax/Bcl-2 protein ratio (**Figure 2E**), while hepatocytes immunopositive to cleaved Cas-3 were rare in both groups, although they were detected slightly more frequently in the diabetic mice (**Figure 2F**).

### 3.3 Oxidative damage, lipid peroxidation and ferroptosis in liver

**Figure 3:**
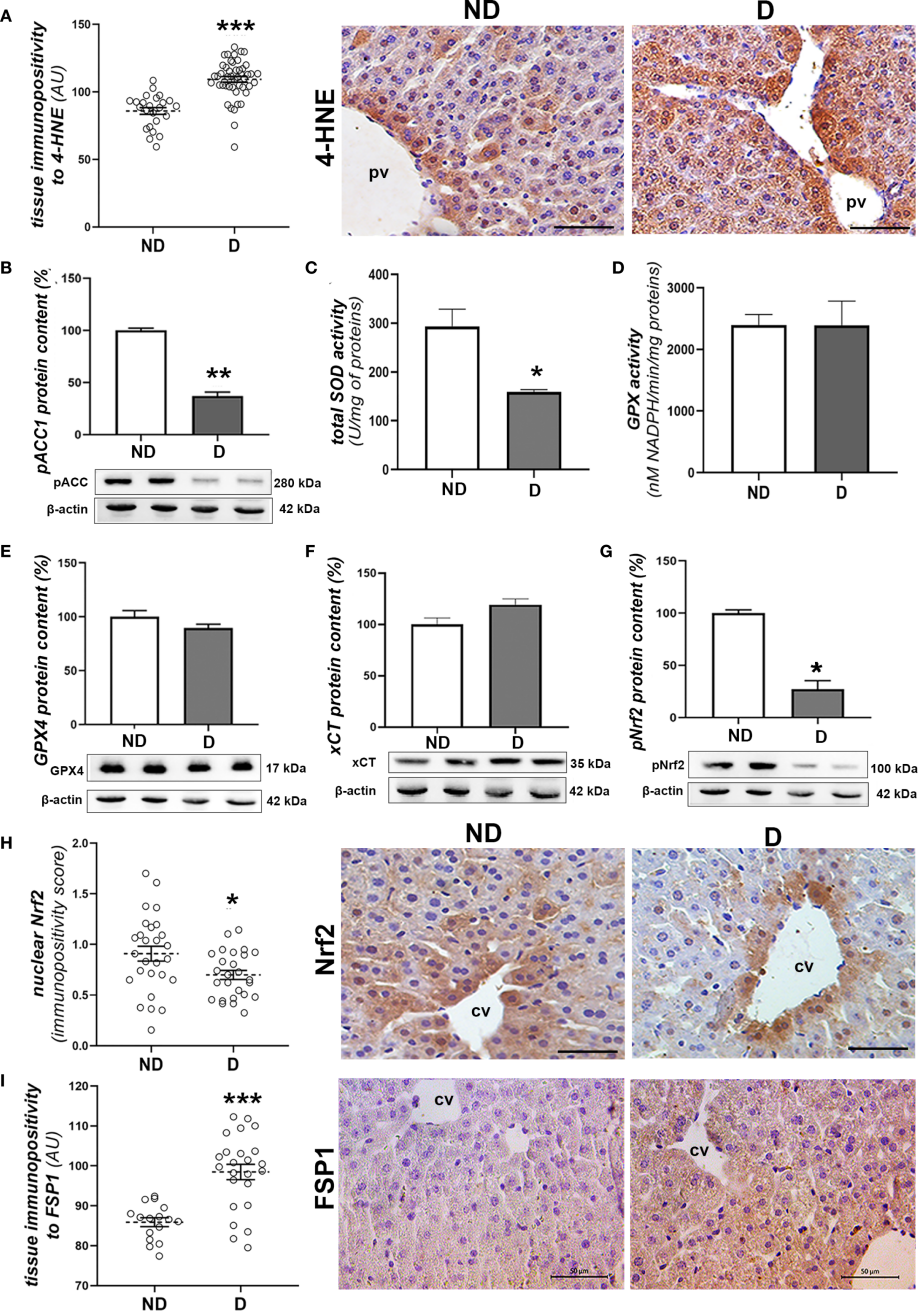
Prooxidative and ferroptotic parameters in the liver of diabetic NOD mice. **(A)** Tissue immunopositivity and immunohistochemical detection of 4-HNE, **(B)** protein content of pACC1; **(C)** total SOD and **(D)** GPX activity; protein contents of: **(E)** GPX4, **(F)** xCT and **(G)** pNrf2; **(H)** nuclear immunopositivity and immunohistochemical detection of Nrf2 and **(I)** tissue immunopositivity and immunohistochemical detection of FSP1. All graph values are mean ± SEM. Statistical significance compared to the ND group: *p<0.05, **p<0.01, ***p<0.001. Original magnification and scale bar: **(A, H, I)** x40, 50 μm. Markings on the micrographs: cv, centroportal vein; pv, portal vein.

To investigate signs of oxidative damage, including signs of lipid peroxidation, we performed immunohistochemical detection of 4-HNE protein adducts and determination of pACC1 protein levels and total SOD activity. The immunopositivity of 4-HNE was significantly increased in liver tissue of diabetic mice compared to non-diabetic mice (p<0.001; **Figure 3A**), which is consistent with the decreased protein level of pACC1 (p<0.01; **Figure 3B**). In parallel, the activity of total SOD decreased (p<0.01; **Figure 3C**), while the total activity of GPX was not altered compared to the non-diabetic mice (**Figure 3D**), nor was the protein content of GPX4 and xCT (**Figures 3E, F**). However, the protein level of activated (phosphorylated) Nrf2 (pNrf2) was significantly lower in the diabetic animals (p<0.05; **Figure 3G**), which was confirmed microscopically by the lower nuclear translocation of Nrf2 in the hepatocytes (**Figure 3H**). Immunohistochemically, increased expression of FSP1 was detected in the hepatocytes of the early diabetic NOD mice compared to the non-diabetic animals (p<0.001; **Figure 3I**).

**Figure 4:**
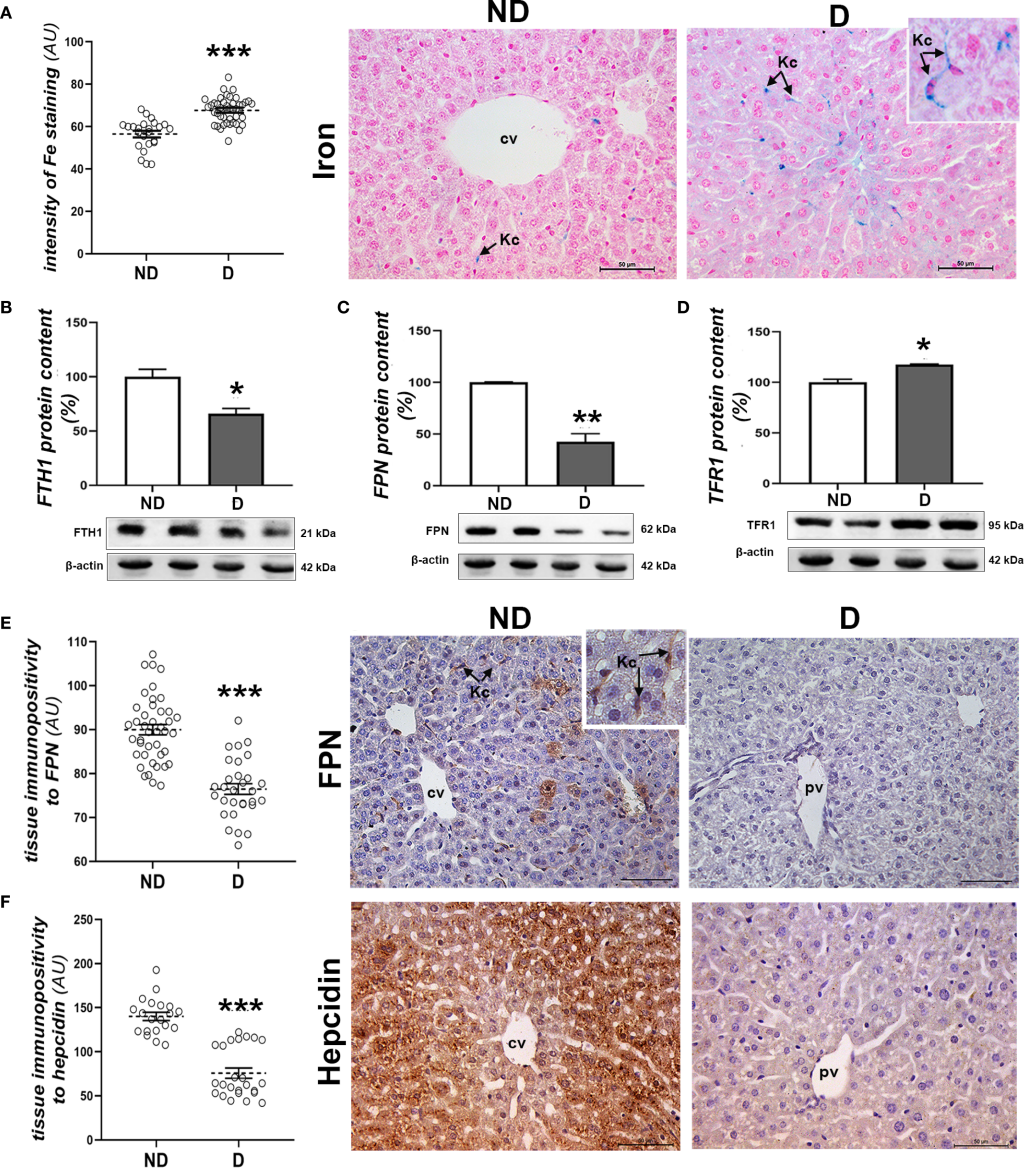
Parameters of iron homeostasis in the liver of diabetic mice. **(A)** Pearl’s iron staining and signal quantification in the tissue; protein levels of **(B)** FTH1, **(C)** FPN and **(D)** TFR1; tissue immunopositivity and immunohistochemical detection of **(E)** FPN and **(F)** hepcidin. All graph values are mean values ± SEM. Statistical significance compared to the ND group: *p<0.05, **p<0.01, ***p<0.001. Original magnification and scale bars: **(A, E, F)** x40, 50 μm. Markings on the micrographs: cv, centroportal vein; pv, portal vein; Kc, Kupffer cells.

In order to further investigate the possible involvement of perturbations in iron metabolism in the liver, an analysis of the accumulation of labile iron was carried out. Histochemical detection of iron (Fe^3+^) ions in the liver of diabetic NOD animals showed an increased iron load in hepatocytes and abundant Kupffer cells compared to the non-diabetic animals (p<0.001; **Figure 4A**). Expression of proteins involved in iron transport was also altered, as the protein levels of FTH1 (p<0.05; **Figure 4B**) and FPN (p<0.01; **Figure 4C**) were decreased and that of TFR1 was increased (p<0.05; **Figure 4D**). Immunohistochemically, lower expression of FPN was detected in both hepatocytes and Kupffer cells in the liver of diabetic NOD animals (p<0.001; **Figure 4E**). Immunohistochemical analysis of hepcidin, an important hormone regulating systemic iron metabolism, revealed a significant decrease in the expression of this protein in the hepatocytes of the diabetic NOD mice (p<0.001; **Figure 4F**).

### 3.4 Early histopathological changes in the kidney of diabetic mice

**Figure 5:**
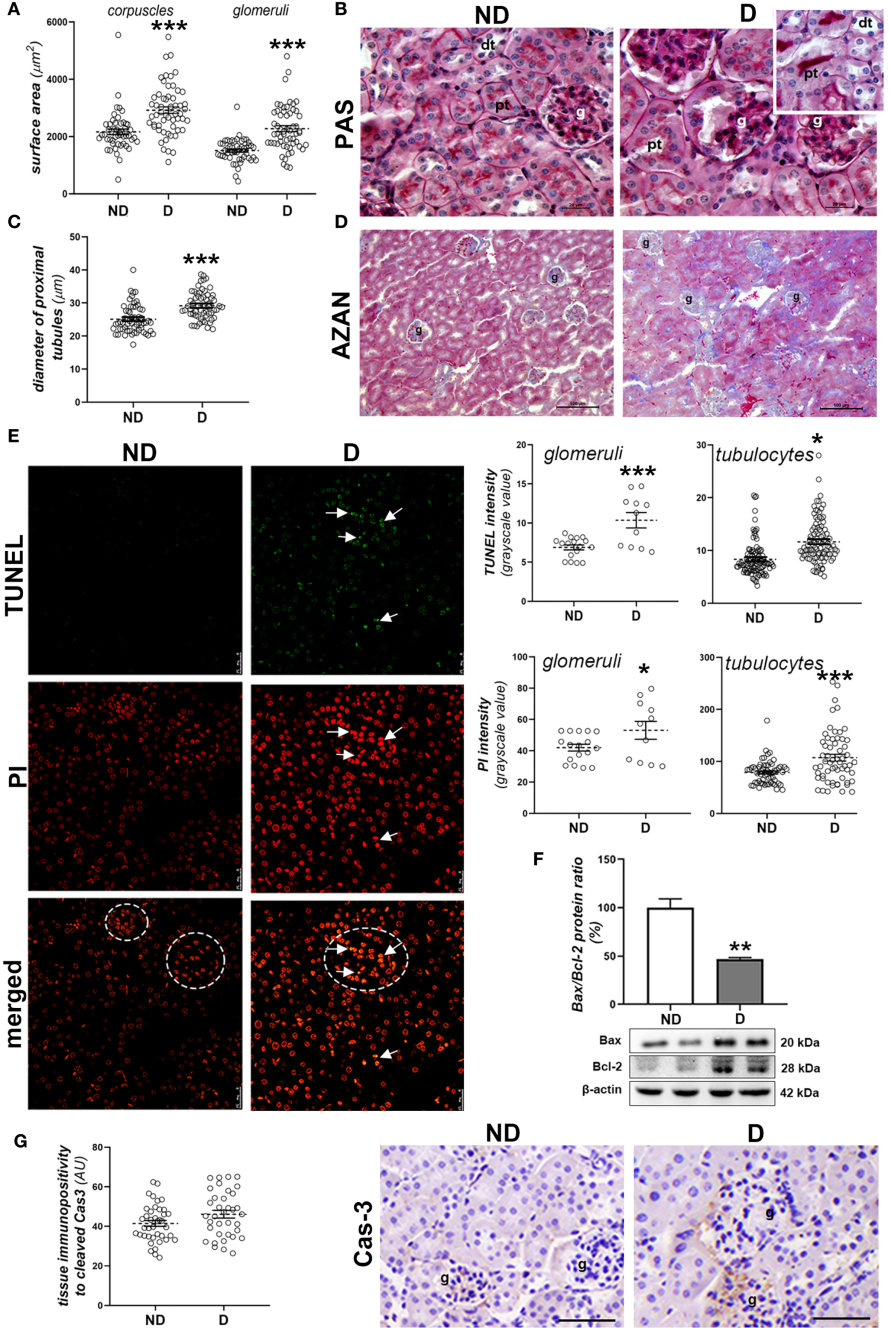
Early diabetic histopathological changes, cell death and damage in the kidney of NOD mice. **(A)** Morphometric measurement of surface area of corpuscles and glomeruli (µm^2^); **(B)** PAS staining (magenta) of renal tissue; **(C)** diameter of proximal tubules (µm); **(D)** collagen detection (blue) in the renal cortex of non-diabetic (ND) and diabetic animals **(D)** - AZAN staining; **(E)** TUNEL and PI staining and subsequent quantification of fluorescence signals in glomeruli (circled) and cortical tubulocytes; **(F)** comparison of renal Bax/Bcl-2 ratio (%); **(G)** tissue immunopositivity and immunohistochemical detection of cleaved caspase-3 (Cas-3). All graph values are given as mean ± SEM. Statistical significance compared to the ND group (*p<0.05, **p<0.01, ***p<0.001). Markings on the micrographs: g, glomerulus; pt, proximal tubule; dt, distal tubule; white arrows, late apoptotic nuclei. Original magnification and scale bar: **(B)** x100, 20 μm; **(D)** x20, 100 μm; **(E)** x63, 25 μm; **(G)** x40, 50 μmx100, 20 µm;.

Within the renal tissue, the most obvious changes were seen in the cortex, including hypertrophy of both the corpuscles and glomeruli (**Figure 5A**; p<0.001), deformation of the glomeruli, the formation of casts (**Figure 5B**) and fibrotic changes around the renal corpuscles and tubules (**Figure 5D**). In the cortical tubules, signs of detachment of the tubulocytes from the basal lamina (**Figure 5B**, insert) and dilatation of the proximal tubules were observed in the diabetic NOD animals (p<0.001; **Figure 5C**).

When analyzing cell death and cell damage in the kidney of diabetic NOD mice, more nuclei which were stronger positive to PI or TUNEL staining were detected in both glomeruli and cortical tubules of diabetic animals (**Figure 5E**, PI staining: p<0.001 (glomeruli), p<0.05 (tubulocytes); TUNEL staining: p<0.05 (glomeruli), p<0.001 (tubulocytes)). Nuclei which were strongly positive to both TUNEL and PI staining were seen mostly within glomeruli. The Bax/Bcl-2 ratio was lower in the kidneys of diabetic NOD mice (**Figure 5F**; p<0.01), while immunopositivity to cleaved Cas-3 was low in the renal cortex of both ND and D animals (**Figure 5G**).

### 3.5 Oxidative damage, lipid peroxidation and ferroptosis in kidney

**Figure 6:**
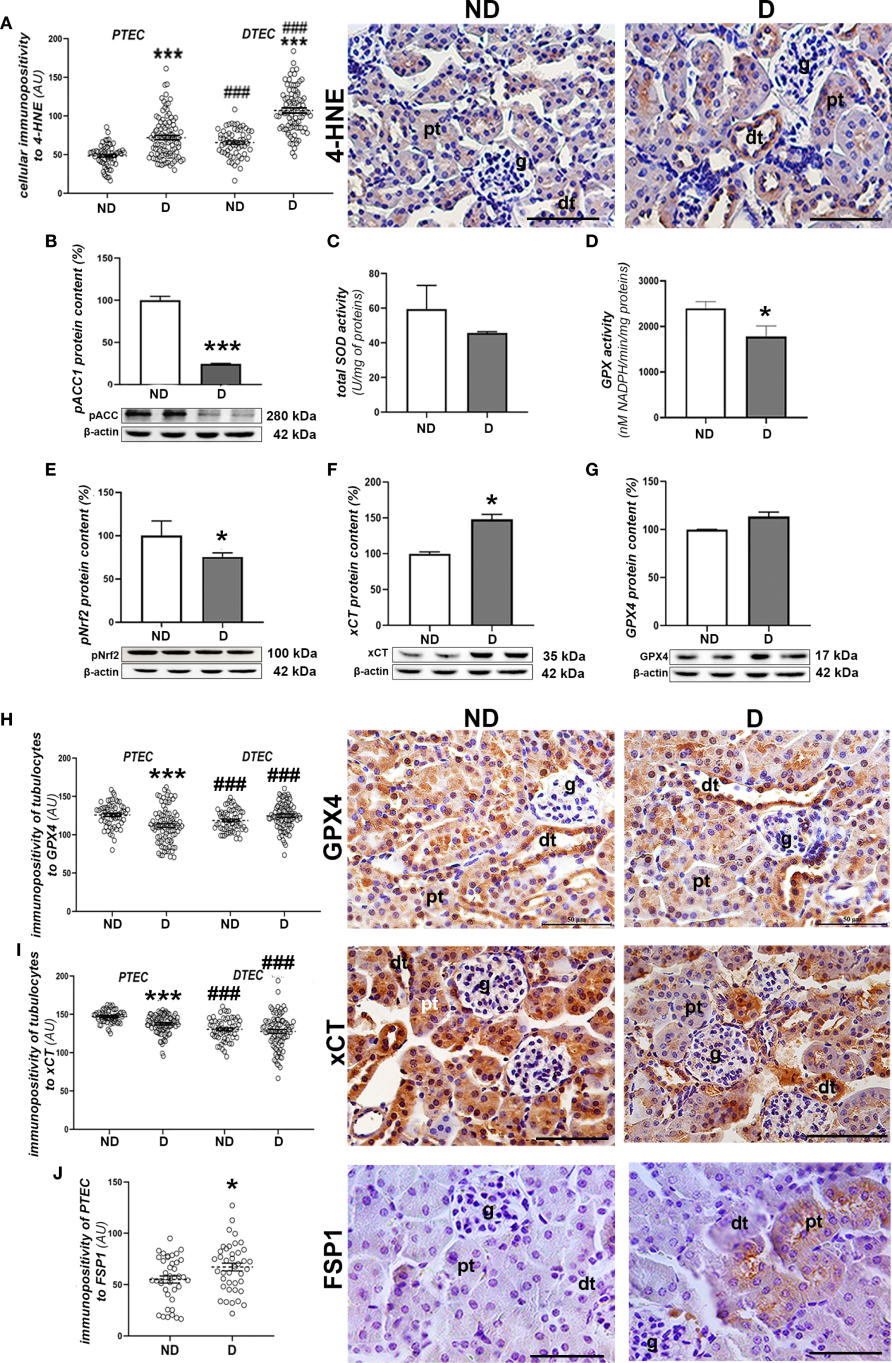
Prooxidative and ferroptotic parameters in the kidney of diabetic NOD mice. **(A)** Quantification of immunopositivity of proximal (PTEC) and distal tubulocytes (DTEC) and immunohistochemical detection of 4-HNE; **(B)** protein level of pACC1; **(C)** total SOD and **(D)** GPX activity; protein contents of: **(E)** pNrf2; **(F)** xCT and **(G)** GPX4; immunopositivity of tubulocytes and immunohistochemical detection of: **(H)** GPX4, **(I)** xCT and **(J)** FSP1. All graph values are given as mean ± SEM. Statistical significance compared to the ND group (*p<0.05, ***p<0.001); statistical significance compared to the PTEC of the same experimental group (^###^p<0.001). Markings on the micrographs: g, glomerulus; pt, proximal tubule; dt, distal tubule. Original magnification and scale bars: x40, 50 μm.

Examination of signs of oxidative injury, including signs of lipid peroxidation, revealed greater 4-HNE immunopositivity (**Figure 6A**) in the proximal (p<0.001) and distal tubules (p<0.001) of renal tissue from diabetic NOD mice compared to non-diabetic mice. No evidence of increased 4-HNE levels was found in the glomeruli. In addition, the protein content of pACC1 was decreased in diabetic animals (p<0.01; **Figure 6B**). At the same time, SOD activity stayed unchanged (**Figure 6C**), while GPX activity was decreased compare to non-diabetic mice (p<0.05; **Figure 6D**). At the tissue level, the protein content of pNrf2 was slightly decreased in the diabetic mice (p<0.05; **Figure 6E**) while the protein content of xCT was slightly higher (p<0.05; **Figure 6F**) or remained unchanged in the case of GPX4 (**Figure 6G**). Immunohistochemically, a decreased positivity of the proximal tubular epithelial cells (PTECs) to GPX4 (p<0.001; **Figure 6H**) and xCT (p<0.001; **Figure 6I**) was noted, while it remained high in the distal tubular epithelial cells (DTECs) of the diabetic mice. In contrast to GPX4 and xCT, the immunopositivity of PTECs to FSP1 was increased in the diabetic animals (p<0.05; **Figure 6J**).

**Figure 7:**
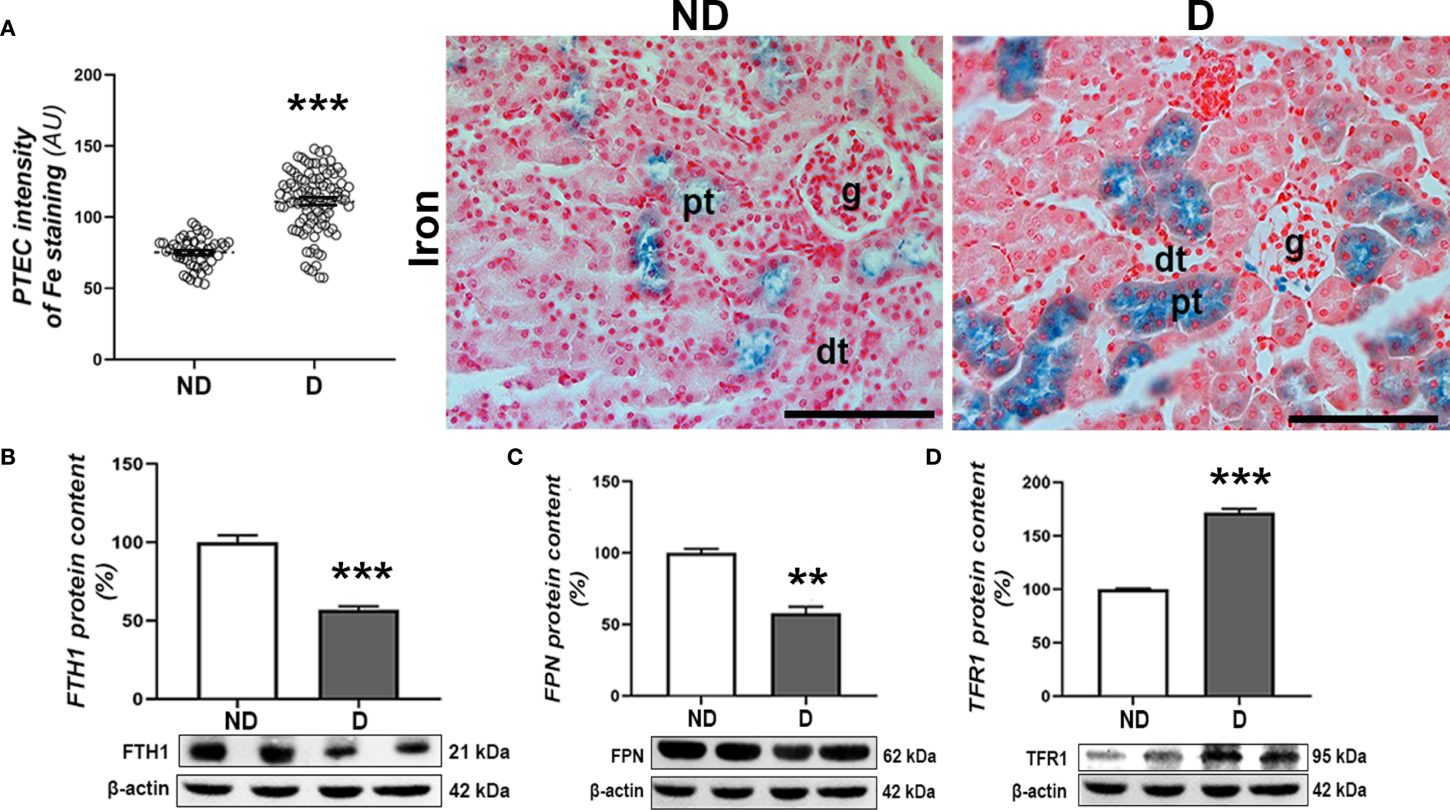
Renal accumulation of iron in diabetic NOD mice. **(A)** Pearl’s staining – detection of Fe^3+^ ions and intensity of Pearl’s staining signal in proximal tubulocytes (PTEC). Protein levels of **(B)** FTH1, **(C)** TFR1 and **(D)** FPN in kidney tissue. All graph values are given as mean ± SEM. Statistical significance compared to the ND group (**p<0.01, ***p<0.001). Original magnification and scale bar: x40, 50 μm.

The histochemical detection of iron (Fe^3+^) ions in the kidney tissue revealed that labile iron mainly accumulates in the proximal tubules of both experimental groups. Nevertheless, a higher accumulation of iron was observed in the diabetic animals (p<0.001) (**Figure 7A**). This is accompanied by a decrease in FTH1 (p<0.001) and FPN protein content (p<0.01) and an increase in TFR1 protein content (p<0.001) in the renal tissue of the diabetic animals compared to the non-diabetic animals (**Figures 7B–D**).

## 4 Discussion

This study demonstrated early disturbances of iron metabolism and iron accumulation in the liver and kidney of NOD mice with spontaneously developed diabetes. This was associated with lipid peroxidation in both organs, but the defense mechanism against ferroptosis including the Nrf2/GPX4/xCT axis was more impaired in the kidneys, more specifically in the proximal tubules. On the other hand, the involvement of apoptosis in these organs seems to be less obvious in early-stage diabetes, as no cleaved Cas-3 immunopositivity or increased Bax/Bcl-2 ratio were detected.

Renal pathological changes in mouse models of diabetes are known to involve damage to the renal corpuscles, including expansion of the mesangial matrix, glomerular hypertrophy and thickening of the glomerular basement membrane. These changes are described as mild and correspond to classes I and IIa of DN in humans (31, 32). However, there is no detailed information in the literature on the pathological changes in the tubule cells of NOD mice and the underlying mechanisms, particularly related to lipid peroxidation and ferroptosis, although the results of other models indicate that these structures are involved in the initial pathological changes in DN development (26, 32). Our study showed impaired kidney function (increased serum levels of urea and creatinine) and initial histopathological changes in the renal cortex, including mild fibrosis, hypertrophy and deformation of renal corpuscles, dilatation of proximal tubules and detachment of tubulocytes. Analysis of cell death parameters revealed increased DNA condensation and fragmentation in both glomeruli and tubules, although cells with nuclei strongly positive to both PI and TUNEL (i.e. late apoptotic) were rare, especially among tubulocytes during this phase of diabetes in NOD mice. On the other hand, proferroptotic events were strongly detectable in the cortical tubules, especially in the PTECs.

Increased level of lipid peroxidation was observed in both PTECs and DTECs from the early diabetic NOD mice, as reflected by increased production of 4-HNE, although the increased iron ion accumulation was only seen in the PTECs. This is supported by the decreased pACC1 protein content in the kidneys of the diabetic NOD mice, indicating the accumulation of the major sources of lipid peroxides, polyunsaturated fatty acids (PUFAs). Acetyl-CoA carboxylase 1 (ACC1) is the first rate-limiting enzyme in fatty acid synthesis, including long-chain PUFAs, and its phosphorylation suppresses the lipid biosynthetic pathway (33). The increased ROS content, especially H_2_O_2_, enhances the Fenton reaction and increases the oxidation of PUFAs of membrane phospholipids. Labile iron ions are the main players in the process of lipid peroxidation. Their overload detected in the PTECs of diabetic NOD mice speaks in favor of their increased glomerular filtration and reabsorption, which occurs predominantly in the proximal tubules (34). This appears to be related to altered protein expression of key players in iron homeostasis, including TFR1, which is involved in iron reabsorption from the tubule lumen, FTH1, which is responsible for cellular iron sequestration, and FPN, which exports iron from tubulocytes and returns it to the circulation. Elevated TFR1 protein levels in the kidneys of diabetic mice are also indicative of increased iron filtration, which is increased in diabetes, as previously shown (35, 36). The decreased levels of FTH1 and FPN suggest an impaired ability of PTECs to store and recycle the excess iron ions in diabetic kidneys (37). Overall, the increased lipid peroxidation associated with the accumulation of iron in PTECs suggests that these cells are susceptible to iron-catalyzed, ROS-mediated damage in the early stages of diabetes (38, 39). This is partly due to direct glucotoxicity, as hyperglycemia attacks these cells from both the basolateral and luminal surfaces, which is due to increased glomerular filtration of glucose. The increased filtration and reabsorption of excess glucose consequently increases OXPHOS and superoxide production (40). Furthermore, the increased glucose uptake leads to the activation of NADH oxidase as an additional source of ROS (41).

Reduced GPX activity detected in renal tissue of diabetic NOD mice suggests impaired protection against ROS, which could increase oxidative pressure in PTECs. Furthermore, impairment of GSH-dependent antiferroptotic defense in PTECs was detected in diabetic mice, as a decrease in GPX4 and xCT immunopositivity of these cells and reduced tissue levels of pNrf2 were found. On the other hand, the increased FSP1 immunopositivity in PTECs from early diabetic NOD mice suggests the involvement of the alternative GSH-independent antiferroptotic pathway in the clearance of lipid peroxides. FSP1 stimulates the reduction of CoQ10 (ubiquinone) to CoQ10H2 (ubiquinol), an antioxidant molecule directly involved in scavenging free radicals to prevent lipid peroxidation, and indirectly through the regeneration of another antioxidant, α-tocopherol (42–44). However, since peroxidative damage has occurred, the GPX4-dependent pathway appears to be more important in protecting these cells from this type of impairment.

Interestingly, lipid peroxidation in the DTECs does not appear to rely on iron-based redox mechanisms, as the increased levels of lipid peroxidation were not associated with either the increased iron accumulation or reduced GPX4 and xCT immunopositivity. Although the formation of lipid peroxides can occur via non-enzymatic, redox-driven mechanisms, enzymatic pathways also play a crucial role (45). In particular, enzymes that metabolize arachidonic acid and PUFAs— with lipoxygenases (LOXs) as central regulators—may account for the observed lipid peroxide accumulation in DTECs, especially since LOX expression and activity are enhanced under hyperglycemic conditions (46). Furthermore, FSP1 immunopositivity was not strong in either normoglycemic or hyperglycemic NOD mice. It is possible that DTECs possess better capacity than PTECs to respond to (lipid per-)oxidative pressure and resist ferroptosis, given their consistently higher GPX4 and xCT protein levels than PTECs under both normoglycemic and hyperglycemic conditions. This interpretation aligns with reports that DTECs are less sensitive to cell death and damage compared to PTECs by synthesizing several anti-apoptotic and anti-necrotic proteins as well as anti-inflammatory, repair and survival factors with autocrine and paracrine function (47). Further evidence for this resilience comes from studies in diabetic settings, where distal tubules did not exhibit accumulation of receptors for advanced glycation end products (AGE), oxidative derivatives due to hyperglycemia (48). Collectively, these findings support the notion of relatively preserved distal tubule function in diabetes, consistent with previous reports (48, 49).

Overall, the results of the present study suggest that diabetic renal pathology in the early phase of spontaneously developing diabetes is associated with a proferroptotic phenotype of PTECs, which appears to be the main mechanism of renal injury in the initial phase of DN pathogenesis. This is consistent with other studies showing that tubular alterations precede the development of glomerulopathy in DN (48). Our results are consistent with data from previous studies in STZ-induced diabetes and in *db/db* mice, as well as an *in vitro* study in PTECs under diabetic conditions, showing ferroptosis as a major mechanism of cell death in the kidney (5). The main difference between our study and the studies based on chemically induced diabetes lies in both timing and tissue specificity. The key difference is that, unlike chemically induced diabetes, the ferroptotic phenotype was examined after prolonged hyperglycemia (8–12 weeks) and in whole-kidney homogenates (50, 51), whereas our study specifically addressed defined renal compartments.

The link between hyperglycemia and the ferroptotic phenotype likely involves additional metabolic components, particularly inflammation. The diabetic milieu activates inflammatory cascades that affect numerous cellular signaling pathways, including those regulating iron metabolism and lipid peroxidation, which ultimately converge in ferroptosis (52). Ferroptotic tubular cells release damage-associated molecular patterns (DAMPs) and oxidized lipid mediators that activate innate immune pathways, thereby exacerbating renal inflammation (53, 54). This inflammatory milieu recruits macrophages, increases cytokine production and promotes fibroblast activation, leading to tubular–interstitial fibrosis and progressive renal dysfunction. Inhibition of ferroptosis using small-molecule inhibitors or iron chelators has been shown to attenuate tubular injury, reduce inflammatory markers, and limit fibrosis in diabetic kidney models. This supports ferroptosis as a mechanistic link between metabolic stress, inflammation, and disease progression in the diabetic kidney (55, 56).

With respect to the liver of NOD mice in the early stages of diabetes, our study showed signs of pathology, including increased ALT activity, hepatocyte hypertrophy, decreased glycogen accumulation, mild fibrosis and an increased number of Kupffer cells. Increased condensation and mild fragmentation of nuclear DNA were observed within the hepatocytes. Further analyses showed signs of oxidative stress and lipid peroxidation in the liver tissue of the early diabetic NOD mice, including decreased activity of SOD, accumulation of 4-HNE and increased pACC1 levels. As for the labile iron pool, we observed a significant accumulation of Fe^3+^ ions in the hepatocytes and Kupffer cells of the hyperglycemic NOD mice. According to our results, this is related to disturbances in the internalization, intracellular storage and export of iron, as evidenced by an increased protein level of TFR1 and a reduced level of FTH1 and FPN. FPN is known to be highly expressed in cells involved in iron turnover (uptake, storage and re-utilization), such as duodenal epithelial cells, hepatocytes and reticuloendothelial macrophages (including Kupffer cells) (reviewed in (57)), as we have shown here in both liver cell types from normoglycemic NOD mice. Suppression of FPN has been suggested to be a factor in iron overload in an *in vitro* study of insulin resistance in primary hepatocytes (58). The decrease in FPN levels in hepatocytes and Kupffer cells in diabetic mice clearly indicates its impaired systemic physiological function in iron efflux from these cells, which, together with the changes in TFR1 and FTH1 levels, is involved in the accumulation of labile iron. Data from the literature suggest that iron accumulation in Kupffer cells (probably due to phagocytosis of dead hepatocytes and red blood cells (59)) leads to advanced liver disease, including fibrosis (60), which we also observed here in the early phase of diabetes in NOD mice.

Regarding the Nrf2/GPX4/xCT axis, only alterations in the translocation of Nrf2 into the nuclei of hepatocytes and in the tissue level of pNrf2 protein were detected, indicating reduced Nrf2 activity in the liver of early diabetic NOD mice. At this stage, protein levels of GPX4 and xCT remained unchanged, suggesting the involvement of additional mechanisms to regulate their expression in early diabetes in NOD mice, which remain to be explored. Interestingly, in our previous studies of chemically-induced diabetes, Nrf2 inactivation was accompanied by a pronounced downregulation of most GSH-related enzymes, including GPX, as well as reduced xCT expression and/or activity after three and six weeks of hyperglycemia (4). In contrast, the present study demonstrates activation of an alternative GSH-independent antiferroptotic pathway in hepatocytes, as evidenced by increased FSP1 expression immunohistochemically. This suggests that FSP1 may contribute to the clearance of lipid peroxides from hepatocytes in the early stages of diabetes.

There is increasing evidence that iron and glucose homeostasis are tightly regulated at both systemic and cellular levels (61). While in T2D excessive systemic iron levels have been shown to play a role in the pathogenesis of the disease mediated by both β-cell damage and insulin resistance (62), its role in T1D is not so clear. According to our results in NOD mice, the accumulation of iron in the liver is a consequence of the diabetic state. Loss of insulin signaling, both due to insulin resistance (in T2D) and insulin deficiency (in T1D), could increase iron overload (63). One of the possible explanations could be the decreased production of the peptide hormone hepcidin by the hepatocytes, as shown here and in previous studies (63). Hepcidin negatively regulates iron homeostasis as it suppresses intestinal absorption and release of iron from macrophages, thus lowering circulating iron levels (64). In addition, increased iron storage in hepatocytes and cells of the reticuloendothelial system has been shown to lead to anemia in patients with T1D (65–67). The main cause of the decrease in hepcidin synthesis in the liver is the loss of insulin signaling, which leads to increased serum iron level and increased iron content in the liver, both of which have been shown to be restored by insulin therapy (63).

Overall, our results show that the first pathological changes in organs affected by hyperglycemia occur soon after the onset of T1D in untreated female NOD mice, with signs of damage in both the liver and kidney. Disturbances in iron homeostasis and lipid peroxidation appear to underlie these early pathologies in NOD mice, suggesting that the same cellular mechanisms drive iron overload in both organs. This underscores the systemic dysregulation of iron metabolism in T1D while revealing organ-specific differences: antiferroptotic defense is less effective in proximal tubulocytes and favors a ferroptotic phenotype in the diabetic kidney. These results suggest that antiferroptotic strategies are promising approaches for early intervention in diabetic kidney disease. Such strategies may include established pharmacologic inhibitors of ferroptosis (lipid peroxide scavengers, iron chelators, or mitochondria-targeted antioxidants) as well as genetic interventions that enhance antiferroptotic genes or suppress proferroptotic genes. In addition, our results emphasize the importance of exploring the antiferroptotic potential of novel antidiabetic agents, some of which are currently under investigation in our laboratory.

Despite the strengths of our study, certain limitations should be acknowledged. First, all experiments were performed in female NOD mice, a model frequently used to investigate the development of T1D (REFS) and its metabolic complications (10, 11, 23, 68, 69). This may limit the applicability of our results to both sexes in humans. Female NOD mice develop T1D earlier and with higher incidence than males, exhibit higher insulin sensitivity and develop insulitis more rapidly (70, 71). These sex-specific differences become more pronounced after sexual maturation, highlighting the influence of sex hormones on the development and progression of T1D. Indeed, the literature indicates that sex hormones strongly influence β-cell protection, gut microbiota composition, and immune responses, with androgens contributing to relative protection in males (72, 73). Moreover, sex-specific differences in disease progression and associated pathologies have been documented (72) and cannot be excluded. Future studies including both sexes are therefore needed to test the applicability of our results on a broader basis.

A second limitation is the lack of pharmacologic inhibition of ferroptosis. While our conclusions are supported by microscopic and molecular evidence consistent with proferroptotic events described by other groups, definitive functional validation typically requires ferroptosis inhibitors such as ferrostatin-1 or liproxstatin-1. In our previous work, ferrostatin-1 was able to attenuate pancreatic and liver injury in STZ-induced diabetes (3, 4, 6), and ongoing studies in NOD mice are testing liproxstatin-1 to further confirm antiferroptotic effects.

Finally, the lack of a control group without the relevant genetic mutations limits the specificity of attributing the observed disorders solely to the development of diabetes. Genetic and phenotypic variability may contribute to immune alterations, autoimmune processes and the timing of diabetes onset and progression. The inclusion of additional control groups in future studies will be important to strengthen the attribution of the observed changes specifically to diabetes. Efforts to this end are currently underway.

## Data Availability

The raw data supporting the conclusions of this article will be made available by the authors, without undue reservation.
